# Assessment of Heavy and Toxic Metals in the Blood and Hair of Saudi Arabia Smokers Using Modern Analytical Techniques

**DOI:** 10.1155/2019/7125210

**Published:** 2019-06-04

**Authors:** Majed Alrobaian, Hassan Arida

**Affiliations:** ^1^College of Pharmacy, Taif University, 888-Taif, Saudi Arabia; ^2^Hot Laboratories Center, Atomic Energy Authority, 13759-Cairo, Egypt

## Abstract

Epidemiological studies on the heavy and toxic metal content in the human blood and hair of some smokers from Saudi Arabia were carried out by modern analytical techniques. The levels of some selected heavy and toxic metals (e.g.; Hg, Pb, Cd, As, Se, Mn, Zn, Ni, and Cr) were determined using inductively coupled plasma-atomic emission spectrometer (ICP-AES). Prior to the analysis, the blood and hair samples of Saudi Arabia smokers were collected, treated, and digested by microwave digestion system. The number of cigarettes per day as well as the smoking period was taken in consideration in this study. The tested elements concentrations in the investigated smoker blood and hair samples were compared with those obtained from some nonsmoking control samples. The samples were collected from the psychiatric hospital in Taif city after issuing the ethical committee license in this regard. The results obtained from this study represent a very important guide for the antismoking organizations. The assessment of some side effects of the smoking in such studies presents vital challenge for the social antismoking authorities and the stakeholder governments to attain the sustainable investment for their people.

## 1. Introduction

Nowadays the human is exposed to the highest levels of toxic and heavy metals coming from many sources, namely, the burning of coal, natural gas and petroleum, and incineration of waste materials worldwide [1]. Such metals represent a major cause of aging, diseases, and even genetic defects. Therefore, accurate and precise determination of these elements in the human fluids and tissues is extremely important [1–3]. In this context, many analytical approaches have been reported for the determination of heavy and toxic metals in biological samples [1–19]. However, the determination of the toxic heavy metals, minor minerals, and even major elements in the human body is a critical issue in the field of forensic and toxicological clinical chemistry. The major minerals which present in high levels such as Na, K, Mg, and Ca are crucial to the metabolism and activity of the human body. The minor minerals which present in lower levels such as Mn, Fe, Co, Cu, and Zn are also essential as metabolic agents as well as enzymes catalysts. The trace heavy and toxic metals (As, Al, Ag, Se, Pb, Ba, Cr, V, Cd, Hg, Ni, Tl, and Sr) should not be present in the human body [1–3]. Although the human body needs certain minerals to maintain a healthy condition, too much of one mineral may become toxic. Moreover, toxic and heavy metals are highly dangerous even in trace levels and may cause chronic or acute poisoning. In fact, these metals are harmful and have no known function in the human body. The toxicity of these metals can result in many illnesses, namely, reduced or damaged mental and central nervous function, lower energy levels, and damage to blood composition, lungs, kidneys, liver, and other vital organs. Moreover, the long-term exposure to such elements may result in slowly progressing physical, muscular, and neurological degenerative processes that mimic Alzheimer's disease, Parkinson's disease, muscular dystrophy, and multiple sclerosis. Among these elements, cadmium and lead are carcinogenic and the most toxic metals. The cardiovascular effects of such two metals in human come from their association with increased blood pressure [1–3].

The toxic or heavy metals enter the human body through many sources, namely, the houses paint, fish, dental amalgam, farming, mining, and smoking including second hand smoke [1]. Smoking is an important source of exposure to nickel, cadmium, lead, and other toxic metals. The cigarette smoke contains such toxic heavy metals inhaled [1, 19]. Once inhaled through smoking, toxic metals have a long biological life span. Chronic adverse effects on human health may, therefore, in later years result from prolonged intake of such toxic elements, some of which are powerful carcinogens. Based on their extreme toxicity, toxic and heavy metals must be detected at very low levels in the human fluids and tissues [4–18]. In this context, blood and hair are the most suitable human biological samples generally used in such metal analysis. The blood samples have been used to assess the heavy metals levels in human body for several years [1, 2, 4–10]. Due to the partitioning of the toxic and heavy metals in the hair, recently hair samples have been used as good index in such studies [1, 3, 11–18].

Combination of the microwave digestion system with the highly sensitive multielemental technique (ICP‐MS) enables reliable determination of elements, particularly heavy metals, at trace concentration levels. Such combination has been widely used in the simultaneous determination of toxic and heavy metals in human blood and hair [3–5, 8, 11, 17]. Atomic absorption spectrometer (AAS) has also been used in the detection of some minerals and toxic heavy metal in biological samples [2, 13–15, 18]. Anodic stripping voltammetry has been applied in the determination of heavy metals (Cd, Cu, and Pb) in blood [6]. It was reported also that X-ray fluorescence was applied in the measurements of different element in human biological samples [9, 16]. Determination of Cu (II), Fe (II), and Cd (II) in blood samples by solid phase extraction with 2-mercaptobenzimidazole immobilized Amberlite XAD-2 was reported [10]. Measurement of trace metals and transition elements in human hair was performed by high-performance liquid chromatography [12, 13]. The determination of some trace elements in hair samples using capillary electrophoresis has also been demonstrated [13].

Recently, inductively coupled plasma-atomic emission spectrometry technique (ICP-AES) is more commonly used for multielement determinations in the biological samples [7, 17]. Such technique has been widely applied as simultaneous multielements analysis from different samples. Prior to analysis, the organic matter, particularly proteins present in the biological samples are generally removed using the digestion of samples with mineral acids. In this context, the wet digestion protocols of some biological samples using the mineral acids have been demonstrated [2, 3, 11, 18]. The more reliable and efficient microwave digestion techniques using the microwave devices recently developed have been widely applied in the pretreatment of the biological samples [2, 14, 17]. Such techniques provide more efficient method than the conventional wet acid method due to the high recoveries generally obtained from microwave induced acid digestion.

Based on the preceding facts, this project realizes a trial to assess the heavy and toxic metals levels in the human blood and hair of Saudi Arabia smokers using the inductively coupled plasma- atomic emission spectrometer (ICP-AES). Prior to the analysis, the investigated samples were digested using the microwave digestion system.

## 2. Materials and Methods

### 2.1. Sampling

The samples under investigation in this study were collected from Taif city, western province of Saudi Arabia from Jan 15 to Mar 30, 2018. The study protocol was approved by the Medical Ethical Committee of Taif University (approval ref. no. 39-35-0022) and written informed consent was obtained from all volunteers who participated in the study. A total of 43 healthy adult male volunteers from the main laboratory visitors of psychiatric hospital in Taif city at the age of 18 or above were investigated.

Aliquots of 4 mL of whole venous blood sample with lithium heparin anticoagulant were taken from each volunteer using sterilized syringes. Approximately 2 cm of the hair samples was collected from the scalp region using stainless steel scissors. The collected samples were classified according to the type of smoking tobacco, stored at 5°C until digestion and analysis processes.

Questionnaire surveys were performed for all participants under the informed consent. The data collected from the survey are gender, life style, age, educational level, marital status, total monthly income, occupation, smoking habits, smoking period, type of smokes, number of cigarettes per day, current health with regard to chronic diseases, and consequently drugs treatment.

### 2.2. Materials

Doubly distilled water (DDW) obtained from Aquatron (A4000D, UK) was used to prepare standard solutions, dilute samples, and wash all tools and glassware throughout. Nitric acid (69%) and hydrogen peroxide (35% w/v) were purchased from Sigma-Aldrich (Germany) and PanReac AppliChem (Germany), respectively. All glassware and plastic used were cleaned by soaking in dilute HNO_3_, rinsed with DDW water, and air dried before use. The validation measurement of ICP-AES was performed using working calibration solutions of the investigated toxic heavy metal ions (Hg, Pb, Cd, As, Se, Mn, Zn, Ni, and Cr). These solutions were prepared using appropriate stepwise dilutions of certified standard stock solutions (Ultra grade, 1000 *μ*g/mL, 5% HNO_3_, ULTRA Scientific Analytical Solutions, USA).

### 2.3. Instrumentation

Anton Paar (model Multiwave PRO, USA) microwave digestion system (maximum power 1500 watts, maximum pressure 580 psi, and maximum temperature 240°C) equipped with rotor 16 and closed vessel (HF100) of Teflon reaction vessels was used in all the digestion procedures of investigated blood and hair samples. Before each digestion, the reaction vessels were rinsed with 5 mL of concentrated nitric acid and thoroughly washed with doubly distilled water.

The investigated toxic and heavy metals were simultaneously determined using a Thermo Scientific (iCAP 7000 series, USA) inductively coupled plasma-atomic emission spectrometer (ICP-AES) instrument supported by Qtera intelligent scientific data solution (ISDS) software.

The generator was operated at a forward power of 1150 W; the plasma, auxiliary, and nebulizer gas are argon with flow rates of 12.0, 0.5, and 0.5 L/min, respectively. The pump flow rate was 1.5 mL/min. The carrier gas flow rate was optimized to obtain maximum signal-to-background ratios.

### 2.4. Microwave Digestion of the Samples

Prior the metal analysis, each investigated sample (1.0 mL of blood or 0.5 g of hair) was individually digested with 4 mL of nitric acid (69%) and 2 mL of H_2_O_2_ (35% w/v) in the microwave digestion system via temperature ramping (ramped to 200°C for 10 minutes, held for 10 minutes, and then cooled until temperature reached below 40°C). All blood samples were homogenized by vortex for 2 min before pipetting to the digestion closed vessel. Three blank digests were carried in the same conditions. After sufficient cooling of the digestion vessels, the resulting clear digested solutions were quantitatively diluted with doubly distilled water before being analyzed by ICP-AES.

### 2.5. Toxic and Heavy Metals Assessment Using ICP-AES

The investigated toxic and heavy metals were analyzed using ICP-AES under optimized plasma condition in all digested blood, hair samples, and blanks as well. The measured samples were nebulized manually downstream to the plasma and the concentrations were automatically determined using the standard calibration graph prepared in the same plasma conditions. These measurements were performed using axial mode for all detected metal ions. The wave lengths (nm) used in the detection of Cd, Pb, As, Hg, Se, Mn, Zn, Cr, and Ni were 228.802, 220.353, 189.042, 184.950, 196.090, 257.610, 213.856, 283.563, and 221.647, respectively. The ICP-AES instrument was adjusted to measure the samples in triplicate and the correlation coefficient and relative standard deviation were automatically calculated. The determined correlation coefficient and RSD were > 0.99998 and < 2 %, respectively.

## 3. Results and Discussion

The concentration of the tested heavy and toxic metals (Hg, Pb, Cd, As, Se, Mn, Zn, Ni, and Cr) was assessed in the investigated whole blood samples (43) and hair samples (43) of some Saudi Arabia smokers (29, ~67 %) and some nonsmokers volunteers (14, ~33%) using calibrated ICP-AES under the optimized plasma conditions previously mentioned. The smoker subjects were categorized according to the type of smokes into two sets, namely, cigarettes smokers (25) and tobacco pipe smokers (4). Using the standard calibration graph, the measurements were performed in triplicate and the mean was automatically calculated. Prior to the metals analysis, all studied samples were completely digested in sufficient amount of nitric acid (69%, 4 mL) and H_2_O_2_ (35%, 2 mL) using closed vessel automated microwave digestion system. The merits offered by the used digestion method include minimum sample handling steps which helps to minimize the sample contamination as well as allowing high throughput of large number of samples. These improve the overall throughout and the reliability of the analysis protocol. The heavy metals selected to be detected in this study were chosen based on their toxicity as well as their relative abundance in smokes tobacco and consequently in the human body of the smokers. Recently, some epidemiological studies have correlated the high levels of the toxic metals in human body with many dangerous diseases and consequently deaths, namely, neurobehavioral decrements, renal impairment, hypertension, cardiovascular problems, immune dysfunction, and gastrointestinal irritation [20].

### 3.1. Toxic Elements Levels in Human Blood (*μ*g/L)

The investigated heavy and toxic elements (Cd, Pb, Hg, Se, Mn, Zn, Cr, and Ni) were determined in whole blood samples of cigarettes and tobacco pipe smokers of some Saudi male volunteers. For comparison, the tested metals were also measured in whole blood samples of nonsmoker volunteers. The data obtained from the analysis of whole blood samples of 14 nonsmokers subjects were summarized in Table 1. The ages of the subjects were classified to three sets ≤ 25 (1), 26-40 (2), and > 40 years. The concentration level ranges and averages of Cd, Pb, Hg, Se, Mn, Zn, Cr, and Ni were 0.09-2.0 (0.58), 12.6-48.1 (20.9), 0.7-7.1 (2.5), 14.0-66.9 (39.9), 1.1-50.4 (9.9), 2586-4717 (3950), 136.2-223.5 (175.0), and 4.3-36.5 (15.8), respectively. The concentrations of the investigated heavy and toxic metals determined in the whole blood samples of nonsmoker subjects come in agreement with those reported in similar studies [20–24] and are almost below the international tolerance levels as well [20–24]. The levels of such toxic heavy metals in biological specimen such as human blood and hair should be below the international tolerance levels as far as possible. However, environmental pollutants have contributed to introduce these elements into biological systems, and therefore the term of tolerant levels should be used instead of reference values [23]. The results obtained show that the levels of the investigated metals in the nonsmoker human blood reasonably fluctuated within relatively narrow range for a given element. Indeed, such fluctuation comes from the wide variety of related parameters of the human subjects, namely, environment, food, age, culture, and habits [23].

The tested heavy and toxic elements were also assessed in the whole blood samples of 4 pipe smoker Saudi volunteers. The ages (≤ 25, 26-40), period of smoking (4-25 y), and the number of pipes per day (1) of the subjects in this study were considered. The data obtained were presented in Table 2. The level ranges and the averages of Cd, Pb, Hg, Se, Mn, Zn, Cr, and Ni were 0.7-3.6 (1.1), 17.0-42.8 (25.1), 5.7-9.1 (3.7), 17.9-47.4 (30.3), 0.4-33.7 (12.1), 2780-5004 (3811.8), 172.5-238.5 (191.8), and 6.0-13.0 (9.7), respectively. These data were compared with those obtained with the nonsmoker subjects (Table 1). As can be seen from the results presented in Tables 1 and 2, in most cases, the younger subjects have lower levels of the investigated toxic element than those showed with the older subjects in both pipe smoker and nonsmoker. It can be seen also that most of the tested elements levels in the whole blood of the pipe smoker subjects (Table 2) are much higher than those of the corresponding elements obtained with nonsmoker subjects (Table 1). Moreover, the concentration of the tested toxic elements significantly increases with increasing the period of smoking (subject 2, Table 2), which indicates that the inhalation of the tobacco pipe smokes dangerously increases the toxic elements levels in the whole blood of the smoking subjects. The levels of Cd, Pb, Hg, and Ni are higher than those reported in similar studies [21, 23].

Table 3 summarizes the concentration of the investigated toxic elements in the whole blood of 25 cigarettes smoker volunteers. In this study, the age of the subjects (≤ 25 (1), 26-40 (2), and > 40 (3) years), smoking period (1-40 years), and number of cigarettes per day (≤10 (1), 11-20 (2), 21-30(3), and >30 (4)) have been taken into consideration. The concentration ranges and the averages of Cd, Pb, Hg, Se, Mn, Zn, Cr, and Ni were 0.2-6.5 (1.8), 9.6-40.6 (23.2), 0.1-7.2 (2.8), 15.4-69.1 (38.0), 1.2-17.1 (6.5), 2919-5487 (4288), 136.2-233.4 (179.0), and 1.4-3338.9 (164.5), respectively. In order to assess the toxicity introduced by cigarette smoking to the human blood, the obtained results (Table 3) were compared with those obtained for the corresponding elements in the whole blood of nonsmoking subjects (Table 1). As can be seen, all determined toxic elements (except Se and Mn) in the whole blood of smoking subjects showed much higher concentration than those obtained with nonsmoking subjects. Moreover, Ni has extremely high concentrations in whole blood of smoker subjects due to its high abundance in cigarettes tobacco and typically increases in the human blood upon smoking cigarettes. The toxic element levels increase with increasing either the smoking period or the number of smoked cigarettes per day. While the smoking period more effectively affects the toxic element level than the number of cigarettes per day, there are synergetic effects on the toxic elements levels with increasing both of them (subjects 3, 7, and 10). Moreover, the levels of Cd, Pb, Hg, and Ni are higher than those determined in the whole blood of nonsmoker subjects reported in similar studies [21, 23]. The concentration of Ni in whole blood of the smoker subjects significantly increases with increasing either smoking period or number of smoked cigarettes per day to the extent reached more than the international tolerance level (subjects 11,12, and 15, Table 3). This attributed to the high abundance of Ni in cigarettes tobacco reported in similar studies [20–24].

### 3.2. Toxic Elements Levels in Human Hair (*μ*g/kg)

Nine toxic and heavy metals (Cd, Pb, As, Hg, Se, Mn, Zn, Cr, and Ni) were determined in human hair samples obtained from 43 volunteers categorized into three sets, namely, nonsmoker (14), pipe smoker (4), and cigarette smoker (25) subjects. All hair samples were collected, treated, and analyzed under the same conditions and the same plasma parameters as well. The data obtained are collected in Tables 4–6. In this study, hair is used as a biomarker to explore the effect of smoking on the metal ions toxicity introduced to the human body of the subjects under investigation. In this context, the World Health Organization (WHO) has recommended the use of hair analysis for heavy metal testing due to the dependence of heavy elements levels in hair on the environmental exposure, food, culture, and habits, particularly smoking [25]. For this purpose, the concentration of the measured toxic and heavy metals in the human hair of nonsmoker subjects (Table 4) was compared with the corresponding metal levels obtained with hair of pipe smoker (Table 5) and cigarette smoker (Table 6) subjects.

The concentration ranges and averages of Cd, Pb, As, Hg, Se, Mn, Zn, Cr, and Ni determined in the human hair of nonsmoker subjects (Table 4) are 3.2-75.4 (14.0),129.0-2803.9 (619.1), 5.9-121.4 (58.2), 25.5-561.0 (151.7), 117.9-341.7 (243.5), 178.6-1667.1 (777.8), 72067-1479921 (271002), 161.8-2209.0 (1039.0), and 130.1-2365.2 (867.1), respectively. These results come in good agreement with those reported in similar studies and are below the international tolerance levels [20, 25]. The relative fluctuation in data obtained in each investigated element level reflects the different environment, food, culture, age, and habits.

The investigated toxic elements were also determined in hair samples of 4 pipe smoker Saudi volunteers. The data obtained are summarized in Table 5 and compared with those obtained from the analysis of hair samples of the nonsmoker subjects (Table 4). The concentration ranges and averages of Cd, Pb, As, Hg, Se, Mn, Zn, Cr, and Ni are 10.8-61.6 (23.9), 402.6-1035.8 (620.3), 2.6-165.7 (63.2), 25.1-409.7 (147.4), 138.7-393.1 (280.4), 511.9-2672.5 (1093.7), 137095.5-215592.9 (176095.2), 397.7-1061.5 (686.0), and 263.9-1388.1 (841.3), respectively. As can be seen, while the averages of Cd, Pb, As, Se, and Mn are relatively higher than those obtained with hair of nonsmoker subjects (Table 4), the averages of Hg, Mn, Zn, Cr, and Ni are almost having same values with those obtained with the hair of nonsmokers. The results obtained are below the international tolerance levels and agree with those obtained in similar studies [20, 25].

Table 6 summarizes the concentration of the investigated toxic elements in the hair samples of 25 cigarettes smoker volunteers. The concentration ranges and averages of Cd, Pb, As, Hg, Se, Mn, Zn, Cr, and Ni are 4.1-255.5 (48.2), 37.9-11495.0 (1796.2), 20.6-335.6 (77.8), 8.8-396.0 (104.2), 44.2-1201.1 (355.3), 458.9-4068.0 (1427.1), 89402.0-748331.3 (224946.7), 132.4-10935.0 (2769.3), and 35.0-8033.7 (1593.5), respectively. As can be seen, all the investigated toxic elements levels in the hair of cigarette smoker subjects (except Hg and Zn) are significantly higher than those obtained with analysis of hair of nonsmoker subjects as well as being higher than those of pipe smoker subjects. While the average of Pb in human hair is significantly raised (threefold), neither Hg nor Zn averages are affected upon smoking of cigarettes. The results obtained come in good agreement with those obtained in similar studies and the averages of the levels are below the international tolerance levels [20, 25].

On the other hand, in both hair and whole blood analysis of the subjects under investigation, the kinds of tested elements raised upon smoking pipe are different from those increased upon smoking cigarettes. On this context, while the averages of Zn and Hg levels are neither affected upon smoking pipe nor cigarettes, the average of Ni levels is highly increased upon smoking cigarettes and not affected upon smoking pipe. Moreover, the relatively high fluctuation obtained with data of each element obtained with the blood analysis is not present in the case of hair analysis. These findings are attributed to the fact that the origin, manufacturing, and contents of pipe and cigarettes tobacco are completely different.

A comparison of the toxic elements levels in blood and hair of nonsmokers and smokers volunteers was done by calculating the T-test using Microsoft Excel of Office 365. A* p* value less than 0.05 was considered to be significant. The results obtained showed that there were significant differences between the levels of most of the investigated toxic heavy metals in the blood of nonsmokers and smokers subjects (*p*; 0.001-0.05). In addition, there were also significant differences between the levels of most of the investigated toxic heavy metals in the hair of nonsmokers and smokers subjects (*p*; 0.002-0.05). These values of the T test typically confirm our findings previously discussed and indicate that the toxic elements levels in blood and hair of smokers are much higher than those obtained with nonsmokers

## 4. Conclusions

A total of 9 heavy and toxic elements were assessed in hair and whole blood samples of 43 subjects of smoking and nonsmoking of Saudi volunteers. Most of the tested elements levels are increased in both whole blood and hair samples upon smoking of pipe and cigarettes tobacco. Some of these elements reveal levels higher than the international tolerance values, particularly Ni in whole blood of cigarettes smoker subjects. The results obtained presented the chemical toxicity introduced to the human body upon smoking. Such findings will support the antismoking authorities with validated data about the dangerous harmful side effects of smoking, thus helping the stakeholder to explore some of the heavy metal toxicity of smoking.

## Figures and Tables

**Table 1:** Toxic elements levels in nonsmokers human blood (*μ*g/L).

No.	Age^*∗*^	Cd	Pb	Hg	Se	Mn	Zn	Cr	Ni
1	2	BDL	13.7	BDL	23.0	25.4	3765.0	223.5	14.5
2	3	0.5	25.9	BDL	40.2	9.1	4717.5	213.0	13.8
3	2	0.4	28.4	3.2	66.9	7.4	4667.2	156.5	36.5
4	3	2.0	48.1	1.1	35.4	4.2	3618.3	172.6	20.1
5	1	1.4	18.2	0.7	29.0	6.2	3904.9	157.7	9.5
6	1	0.6	18.1	3.3	57.0	2.3	4124.5	182.7	15.6
7	1	0.7	16.9	3.8	42.3	10.0	4106.8	216.3	23.9
8	1	BDL	13.8	3.5	55.5	BDL	3666.6	195.4	10.2
9	1	BDL	21.9	4.9	31.1	BDL	4454.6	206.0	12.7
10	1	0.9	20.9	7.1	46.0	50.4	3497.5	136.3	22.7
11	1	0.1	12.6	2.4	34.4	1.1	4282.8	152.9	BDL
12	1	BDL	19.0	BDL	39.5	16.0	4023.9	141.0	13.9
13	1	0.7	14.2	BDL	14.0	3.0	3891.6	140.5	4.3
14	1	0.7	20.8	5.4	43.8	3.0	2586.2	156.5	7.5

^**∗**^ *Age: 1= ≤25, 2= 26-40, 3= >40 y*.

**Table 2:** Toxic elements levels in pipe smokers human blood (*μ*g/L).

No.	Smo- King^*∗*^	Age^*∗∗*^	Cd	Pb	Hg	Se	Mn	Zn	Cr	Ni
1	14/1	2	0.7	21.7	BDL	17.9	5.3	3475.5	172.5	6.0
2	25/5	2	3.6	42.8	9.0	47.4	9.0	5004.0	238.5	13.0
3	8/1	1	BDL	17.0	BDL	23.6	0.4	3987.3	183.2	10.6
4	4/1	1	BDL	18.8	5.7	32.5	33.7	2780.4	172.9	9.1

^*∗*^ Smoking; period of smoking (y)/rate of smoking (number of pipes per day),

^*∗∗*^ *Age; 1= ≤25, 2= 26-40 y*.

**Table 3:** Toxic elements levels in cigarettes smokers human blood (*μ*g/L).

No.	Smo- King^*∗*^	Age^*∗∗*^	Cd	Pb	Hg	Se	Mn	Zn	Cr	Ni
1	10/3	2	2.2	12.1	1.4	27.9	3.6	5080.5	220.5	17.8
2	20/2	2	1.3	9.6	1.0	25.8	3.9	5053.5	198.0	15.4
3	20/4	2	0.7	21.3	3.3	16.0	7.8	4344.0	228.0	78.0
4	7/1	2	BDL	26.9	4.1	19.8	4.7	4731.0	202.5	15.6
5	40/2	3	0.7	25.7	5.3	22.4	6.5	4111.5	186	13.2
6	15/3	2	2.5	23.8	2.7	34.2	5.3	5205.0	196.5	10.6
7	25/3	3	1.7	40.6	2.2	27.2	BDL	4004.2	169.7	21.5
8	13/2	3	1.8	25.0	7.2	52.5	5.1	4066.3	167.1	17.3
9	30/2	3	2.6	24.8	3.1	29.8	3.6	5008.9	167.4	13.0
10	25/4	3	2.9	21.3	4.5	69.1	4.6	4349.3	136.2	9.8
11	11/4	3	1.7	31.2	5.8	59.3	14.2	4294.5	233.4	3338.9
12	11/2	2	2.0	24.4	2.2	38.0	4.8	3039.0	154.5	266.9
13	30/2	3	6.5	25.4	0.1	39.3	4.6	5487.4	177.3	10.9
14	15/3	2	4.0	35.3	2.9	34.2	5.3	3455.5	199.3	51.1
15	16/4	2	4.9	39.0	1.5	62.0	16.4	4166.7	153.5	94.5
16	10/2	2	4.1	22.3	2.8	67.6	1.9	5219.0	217.9	44.9
17	4/2	1	0.2	16.9	BDL	46.8	13.7	4616.7	190.4	13.7
18	6/3	1	2.6	15.3	0.2	15.4	4.1	4623.2	195.5	15.8
19	1/1	1	0.9	12.5	BDL	43.7	1.8	3424.2	159.4	4.4
20	5/1	1	0.7	9.7	1.3	50.4	8.7	4575.0	145.0	14.5
21	2/1	1	0.2	18.7	2.5	21.1	3.2	3445.4	148.7	7.01
22	1/1	1	BDL	28.1	3.4	35.9	17.1	4522.1	165.2	23.6
23	7/2	1	0.8	20.8	0.3	31.7	11.5	4285.3	152.6	8.6
24	4/1	1	0.2	14.7	5.8	41.5	1.2	2919.1	166.1	1.4
25	4/2	1	BDL	345.0	6.2	38.3	2.9	3173.4	144.7	3.9

^*∗*^Smoking; period of smoking (y)/rate of smoking (number of cigarettes per day),

^*∗∗*^ *Age; 1= ≤25, 2= 26-40, 3= >40 y*.

**Table 4:** Toxic elements levels in nonsmokers human hair (*μ*g/kg).

No.	Age^*∗*^	Cd	Pb	As	Hg	Se	Mn	Zn	Cr	Ni
1	2	6.0	157.9	121.4	25.5	252.4	752.0	115479.9	161.8	130.1
2	3	6.7	214.3	46.4	273.1	286.2	598.8	120714.6	179.8	195.3
3	2	75.4	2803.9	BDL	149.6	165.8	1044.3	86216.1	2209.5	838.2
4	3	5.0	302.9	100.7	561.0	269.9	396.2	155798.5	462.4	618.4
5	1	3.2	129.0	5.9	148.6	117.9	178.6	72067.9	2153.6	201.0
6	1	5.7	363.5	BDL	122.2	251.3	450.5	182640.1	1246.0	296.9
7	1	7.2	418.2	83.5	194.6	203.1	968.2	202796.5	731.1	1986.9
8	1	11.3	371.2	23.0	95.2	284.3	513.3	313091.6	723.8	622.1
9	1	13.2	1234.8	BDL	69.2	176.7	1436.1	1479920.8	2077.5	2365.2
10	1	10.5	297.3	BDL	57.1	196.0	404.5	97978.3	782.3	1841.5
11	1	17.9	792.9	60.0	27.3	341.7	1667.1	145095.0	881.8	317.6
12	1	8.6	319.7	81.8	128.9	241.1	673.1	498220.3	1347.9	1654.3
13	1	22.9	1083.9	32.8	116.4	341.2	1418.4	163349.1	937.0	807.72
14	1	3.2	177.4	26.1	155.2	281.4	387.9	160660.7	652.5	264.6

^*∗*^ *Age; 1= ≤25, 2= 26-40, 3= >40 y*.

**Table 5:** Toxic elements levels in pipe smokers human hair (*μ*g/kg).

No.	Smo King^*∗*^	Age^*∗∗*^	Cd	Pb	As	Hg	Se	Mn	Zn	Cr	Ni
1	14/1^sh^	2	11.0	402.6	165.7	65.9	393.1	598.6	160886.2	397.7	263.9
2	25/^sh^	2	61.6	BDL	BDL	409.7	BDL	2672.5	137095.5	BDL	427.0
3	8/1^sh^	1	12.1	1035.8	21.4	25.1	138.7	591.9	215592.9	1061.5	1388.1
4	4/1^sh^	1	10.8	422.6	2.6	89.0	309.5	511.9	190806.4	598.7	1286.4

^*∗*^Smoking; period of smoking (y)/rate of smoking (number of pipes per day),

^*∗∗*^ *Age; 1= ≤25, 2= 26-40 y*.

**Table 6:** Toxic elements levels in cigarettes smokers human hair (*μ*g/kg).

No.	Smo- King ^*∗*^	Age^*∗∗*^	Cd	Pb	As	Hg	Se	Mn	Zn	Cr	Ni
1	10/3	2	39.5	452.1	31.5	44.7	241.6	458.9	189425.0	132.4	684.1
2	20/2	2	105.6	BDL	BDL	BDL	1201.1	1327.0	97851.5	BDL	1108.2
3	20/4	2	13.6	37.9	32.1	113.7	301.5	602.4	115932.7	198.7	BDL
4	7/1	2	64.6	1594.3	BDL	BDL	79.3	628.5	156509.9	9372.4	2262.0
5	40/2	3	7.2	1247.5	41.7	BDL	240.6	557.9	140960.3	1447.9	661.1
6	15/3	2	91.4	1083.1	BDL	137.5	294.3	1945.1	136082.4	1747.2	807.8
7	25/3	3	7.3	432.7	72.2	25.6	44.2	575.5	89402.0	481.2	187.9
8	13/2	3	28.4	920.9	BDL	76.7	879.8	827.7	294123.4	6507.7	2462.9
9	30/2	3	94.6	1824.8	BDL	BDL	634.0	919.2	200694.8	10935.0	2649.4
10	25/4	3	84.8	4385.8	BDL	BDL	528.3	2523.4	152466.3	7705.5	3673.0
11	11/4	3	72.1	801.0	BDL	294.8	90.8	956.2	136614.6	2658.3	1082.5
12	11/2	2	48.4	835.0	BDL	18.81	181.7	1461.3	529246.1	4654.2	2108.4
13	30/2	3	46.5	1683.3	BDL	BDL	236.9	1718.8	124587.0	7636.0	2402.5
14	15/3	2	255.5	5948.9	55.5	109.9	265.5	4068.0	164403.9	2186.7	1145.1
15	16/4	2	62.3	11495.0	24.8	69.6	175.3	2340.7	326442.8	1246.0	2912.5
16	10/2	2	7.8	706.4	39.6	146.2	305.8	1310.6	180004.8	925.0	233.4
17	4/2	1	12.5	838.5	168.2	8.851	428.8	1734.3	255844.4	1009.4	777.9
18	6/3	1	22.3	512.2	335.6	396.0	1068.6	1163.0	748331.3	1456.0	35.0
19	1/1	1	8.4	278.1	66.9	135.6	231.5	513.9	154201.9	714.5	289.5
20	5/1	1	33.5	1113.4	73.4	23.5	188.9	2007.9	364028.7	925.5	8033.7
21	2/1	1	26.2	541.8	20.6	37.2	271.8	2683.1	165521.6	1340.1	611.6
22	1/1	1	13.3	246.9	36.1	31.5	227.4	1027.4	163442.7	888.7	429.5
23	7/2	1	36.8	5399.4	38.0	147.3	158.1	2116.6	331543.6	840.2	2196.1
24	4/1	1	4.1	183.7	45.6	118.7	335.1	557.3	198630.3	640.3	206.3
25	4/2	1	18.1	545.8	163.2	44.6	272.1	1653.3	207374.4	814.4	1282.5

^*∗*^Smoking; period of smoking (y)/rate of smoking (number of cigarettes per day),

^*∗∗*^*Age; 1= ≤25, 2= 26-40, 3= >40 y*.

## Data Availability

I declare that all the data of the article are available for others. All the data supporting my findings are included in the article.
